# Effects of Glucose Oxidase and Sodium Stearoyl Lactate as a Compound Modifier on Improving the Quality of Wheat Dough and Its Steamed Bread

**DOI:** 10.1002/fsn3.71142

**Published:** 2025-10-27

**Authors:** Yuan‐Bao Jin, Jin‐Ming Tan, Huan Yang, Ping Liu, Hong Wu, Bing Li

**Affiliations:** ^1^ Modern Agriculture and Food College, Ji'an College Ji'an China; ^2^ School of Food Science and Engineering South China University of Technology/Guangdong Province Key Laboratory for Green Processing of Natural Products and Product Safety Guangzhou China; ^3^ Traditional Chinese Medicine Department, Ji'an Central People's Hospital Ji'an China

**Keywords:** compound modifier, dough, rheological properties, steamed bread, water distribution

## Abstract

Modifiers are widely used in flour products; however, a certain modifier often exhibits limited efficacy or requires a high addition amount to achieve the desired improvement. The use of compound modifier (CM) is an alternative strategy to address this problem. In this paper, the effects of glucose oxidase (GOD) and sodium stearoyl lactate (SSL) as a CM on the properties of the dough, as well as the quality of the steamed bread were investigated. The results revealed that CM significantly ameliorated the specific volume (by 9.62%, *p* ≤ 0.05) and texture (by 35.70% for hardness, *p* ≤ 0.05) of the steamed bread, compared with the control group. The CM dough exhibited an increased bound water content (by 21.46%, *p* ≤ 0.05) and improved rheological properties relative to the control. Moreover, CM improves the gluten network in dough by increasing the number of disulfide bonds and strengthening the secondary structure of gluten proteins. These improvements are further supported by microstructural observations of dough and steamed bread using SEM. Both the results of LF‐NMR and sulfhydryl group determination demonstrated a combined improvement effect that occurred between SSL and GOD, which improved the quality of the dough and steamed bread. The results suggest that this CM is very potential in the production of high‐quality flour products.

## Graphical abstract

This study involved developing a compound modifier (CM, SSL + GOD) to enhance the quality of dough and its steamed bread. The results showed that CM significantly improved the dough's water‐holding capacity and rheological properties, enhancing the quality of the final product. This improvement may be due to the synergistic effect of SSL and GOD on the gluten network structure of the dough.

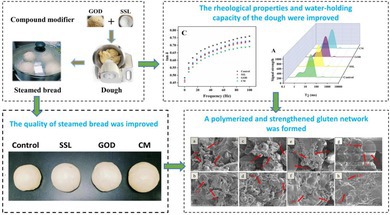

## 1 Introduction

Steamed bread is a traditional fermented food in China, consumed by nearly half of the Chinese population daily (Li, Cao, et al. 2021; Li, Guo, et al. 2021). The main ingredient of the steamed bread is wheat flour, which is widely cultivated and has a high annual yield in China. However, the quality of wheat flour varies greatly and affects the quality of the steamed bread (Guo et al. 2021). Although China has a wide range of wheat varieties, the yield of quality wheat was low. Work has been going on to breed new wheat varieties (Bartkiene et al. 2022) and apply genetic engineering techniques (Cao et al. 2020) for obtaining high quality wheat flour. However, compared to these methods, the use of modifiers to improve the quality of wheat dough is the most common and cost‐effective method. Among the various modifiers used in the flour products, enzyme preparations and emulsifiers are commonly used and proven to be effective (Asghar et al. 2011). Enzyme preparations have attracted more and more attention in recent years due to their advantages of safety, specificity, and catalytic efficiency (Tebben et al. 2018). Meanwhile, emulsifiers serve as conventional improvers in the pasta industry, utilized across diverse applications. However, a single modifier often has defects such as limited effects or a large addition amount to achieve the desired improvement (Cao et al. 2021; Ma et al. 2022), which limits the application of modifiers. Exploring new modifiers or finding efficient ways to use existing ones is a possible strategy to solve this problem, while the latter is simple and more feasible. It has been reported that the combination of glucose oxidase (GOD) and laccase, GOD and pentosanase, amylase and laccase, amylase and protease, and pentosanase and protease exhibited synergistic effects on improving the quality of the bread (Caballero et al. 2007). The combination of *Aspergillus oryzae* S_2_ α‐amylase, ascorbic acid, and GOD showed a comprehensive improvement effect on the properties of the dough and bread (Sahnoun et al. 2016). These results indicate that the combination of multiple modifiers can achieve a complementary effect and reduce their adding dosage.

Our previous research has shown that combining emulsifiers and enzyme preparations significantly improves the quality of dough and dough products (Tan et al. 2023). Following this pattern further may lead to the discovery of new composite improvers. GOD is one of the enzyme preparations that has been widely applied in various fields, such as animal husbandry, winemaking, medicine, and food industry. Studies have reported that GOD can improve the quality of the dough products by promoting protein aggregation, gluten development, and gluten network formation (Niu et al. 2018; Sarabhai et al. 2021). The mechanism by which GOD promotes gluten development is that it can oxidize the sulfhydryl group (‐SH) groups in gluten proteins to form disulfide bonds (‐S‐S‐), thereby increasing the crosslinking degree of the gluten network (Niu et al. 2018). Sodium stearoyl lactate (SSL) is currently the most used anionic food emulsifier and modifier of the dough products in the world. It has been reported that SSL can reduce the hardness and increase the volume of the steamed bread (Tan et al. 2023). In addition, SSL, as an emulsifier, can interact between gluten protein molecules, promoting their interconnection and thus forming a stable gluten network structure (Meng et al. 2019). As mentioned above, the effects of individual GOD and SSL on the improvement of the dough have been reported widely. We hypothesize that GOD and SSL may synergistically enhance the gluten network structure of dough, but their combined effects have not yet been explored.

In our preliminary work, we found that the compound modifier (CM) of GOD and SSL exhibited superior improvement effects on farinograph properties of wheat flour over the single modifier. Subsequently, using the key farinographic parameters water absorption and stability time as representative indicators, response surface optimization was employed to determine the optimal formulation of CM as 30 mg GOD and 140 mg SSL per kilogram of flour (unpublished data). We speculate that CM also has a good improvement effect on wheat dough and its products. Therefore, the study aimed to: (1) examine the impact of the CM on improving the quality of medium‐gluten wheat dough products (steamed bread); (2) assess its effect on enhancing the processing properties of medium‐gluten wheat dough; (3) explore the underlying action mechanisms of the CM. The obtained results could provide some reference on the combination of traditional modifier and enzyme preparation in improving the quality of the flour products.

## 2 Materials and Methods

### 2.1 Materials

Medium‐gluten wheat flour with 15.7% moisture, 12.3% protein, and 0.40% ash was purchased from Yuliang Group Puyang Special Flour Co. Ltd. (Henan, China). High‐activity dry yeast was obtained from Xinliang Liangrun Whole Grain Food Co. Ltd. (Henan, China). GOD (100 U) was acquired from Macklin Biochemical Technology Co. Ltd. (Shanghai China). SSL (99%) was supplied by Yuanye Biological Technology Co. Ltd. (Shanghai China). All other reagents were of analytical grade.

### 2.2 Dough Preparation

Dough preparation was carried out according to our previous method (Tan et al. 2023). 100 g of flour, 53 g of water, and different amounts of modifiers (as shown in Table 1) were mixed in a kneader (PE 4500, Petrus, China) for 7 min. The dough was left to rest for 10 min before further analysis.

**TABLE 1:** Amount of modifiers added to each sample.

Samples	SSL	GOD
Control	0	0
SSL	140 mg	0
GOD	0	30 mg
CM	140 mg	30 mg

*Note:* SSL represents sodium stearoyl lactate, GOD represents glucose oxidase, and CM represents compound modifier.

### 2.3 Steamed Bread Preparation

The steamed bread formulation consisted of 100 g of flour, 53 g of water, 1 g of dry yeast, and different amounts of modifiers (as shown in Table 1). First, all ingredients were blended in the kneader for 7 min and then incubated for 10 min at 25°C. Secondly, the dough was divided into equal 50 g pieces each and shaped into round balls. The dough pieces were then fermented in a constant temperature and humidity box (Yi Heng Shanghai, China) for 1 h (35°C, humidity 85%). Finally, the dough was steamed in an electric steamer (Midea, China) for 30 min, and cooled for 1 h at 25°C before testing.

### 2.4 Low‐Field Nuclear Magnetic Resonance (LF‐NMR)

The water distribution of dough was determined as described by Yang et al. (2021). 10 g of dough was weighed and wrapped in plastic wrap, and then the dough was put into the nuclear magnetic test tube. The transverse relaxation time (*T*_2_) was measured by the NMI20‐040H‐IN LF‐NMR analyzer (Shanghai Newman Electronic Technology Co. Ltd., China). The sampling frequency was 200 kHz, the number of sampling points was 120,022, the sampling time interval was 3500 ms, the echo time was 0.2 ms, the number of echoes was 1000, and the number of scan repetitions was 8.

### 2.5 Rheology Test

The rheological characteristics of dough were determined by using an AR 1500EX rheometer (American TA company, USA). Approximately 5 g of prepared dough was placed in the center of the flat‐bottom testing plate. A 40 mm parallel plate probe was then used to gradually compress the dough until there was a 2 mm gap between the probe and the bottom plate. Any dough extruded around the probe was scraped off with a hard plastic spatula, and edible oil was applied around the probe to prevent the dough from losing moisture during testing. The frequency sweep test was performed within a range of 0.1 to 100 Hz, with the strain amplitude kept constant at 1%.

### 2.6 Steamed Bread Specific Volume Determination

The cooled steamed bread was weighed and its volume was measured by the millet displacement method. The specific volume was calculated by dividing the volume by the weight.

### 2.7 Determination of the Texture of Steamed Bread

A TA.XT.Plus texture tester (SMS, UK) was used to determine the hardness, chewiness, springiness, and resilience of the steamed bread (Li, Cao, et al. 2021; Li, Guo, et al. 2021). The center part of the sample was taken and cut into 15 mm slices. A P36R probe was selected, the steamed bread slice was put on the testing table for the TPA test. Parameter settings: The pretest rate was 1.0 mm/s, the test rate was 1.0 mm/s, the posttest rate was 2.0 mm/s, the compression level was 50%, the induction force was 5 g, and the interval between two compressions was 5 s.

### 2.8 Determination of Free Sulfhydryl Group (‐SH) and Disulfide Bonds (‐S‐S)

The methods for measuring free ‐SH and ‐S‐S bond content were adapted from Liu et al. (2016) and Guo et al. (2021) with some modifications. The dough sample was freeze‐dried for 48 h, then pulverized and sieved through a 100‐mesh screen.

A sample of 75 mg (dry weight) was dissolved and mixed with 1 mL of Tris‐Gly solution (pH 8.0, 5.2 g of Tris, 3.45 g of Gly, and 0.6 g of EDTA in 500 mL of distilled water), then added with 4.7 g of Gu‐HCl and vortexed well. Then, 9 mL of Tris‐Gly solution was added and the mixture was vortexed for 5 min at room temperature. The mixture was then centrifuged at 6000 r/min for 5 min.

For the determination of free ‐SH group, 1 mL of the supernatant solution after centrifugation was taken and mixed with 4 mL of urea/Gu‐HCl solution (8 mol/L, dissolved in 5 mol/L Gu‐HCl/Tris‐Gly solution). The mixture was shaken for 2 h at 25°C, and 0.05 mL of Ellman's reagent (0.1 g of DTNB dissolved in 25 mL of Tris‐Gly solution) was added. The absorbance was measured at 412 nm. 1 mL of distilled water was added to 4 mL of urea/Gu‐HCl solution to serve as a background subtraction.

For the determination of total ‐SH group, another aliquot of 1 mL of the supernatant solution was taken and mixed with 0.05 mL of mercaptoethanol and 4 mL of urea/Gu‐HCl solution. The mixture was incubated for 1 h at 25°C, followed by the addition of 10 mL of 12% trichloroacetic acid, continuing the incubation at 25°C for 1 h and then centrifuged at 5000 r/min for 10 min. The precipitate was washed twice with 5 mL of 12% trichloroacetic acid and dissolved in 10 mL of 8 M urea. Finally, 0.04 mL of Ellman's reagent was added, and the absorbance was measured at 412 nm.

The ‐SH content was calculated as follows:

$$‐\mathrm{SH}\;\left(\text{μmol}/g\right)=73.53\times A_{412}\times D/C.$$

where *A*_412_ is the absorbance value at 412 nm, *D* is the dilution factor (5.02 for free ‐SH group, 10 for total ‐SH group), and *C* is the concentration of the sample (mg (dry)/mL).

The ‐S‐S content was calculated as follows:

$$‐S‐S\;\left(\text{μmol}/g\right)=\left(N_{2}-N_{1}\right)/2.$$

where *N*_1_ is the number of free ‐SH groups, and *N*_2_ is the number of total ‐SH groups.

### 2.9 Fourier Transform Infrared (FTIR)

The secondary structure of gluten proteins was measured by using Tensor‐27 FTIR (Bruker, Germany). The lyophilized sample of dough was mixed with KBr at a ratio of 1:100, and the mixture was ground well in an agate mortar. After pressing into the pellet, the sample was scanned for the full band from 500 to 4000 cm^−1^, the resolution was set to 4 cm^−1^, and the number of scans was 32. The spectra were analyzed using OMNIC and Peakfit 4.12 software. The Gaussian smooth deconvolution and second derivative fitting were used to fit the Amide I band to calculate the distribution of secondary structures of the protein.

### 2.10 Scanning Electron Microscope (SEM)

The microstructures of wheat dough and steamed bread were observed by EVO‐18 SEM (Carl Zeiss, Germany). The dough sample was prepared by pulverizing and sieving the freeze‐dried dough through a 50‐mesh screen. The steamed bread sample was prepared by selecting the free‐falling fragments from the freeze‐dried steamed bread. After vacuum gold spraying, the freeze‐dried samples were detected at room temperature under 30 kV accelerating voltage, and the microstructure was observed at 3.0 k × (dough) and 1.3 k × (steamed bread) magnification, respectively.

### 2.11 Statistical Analysis

The data were analyzed and plotted using SPSS 26 and Origin 2018. The results were expressed as the mean ± standard deviation of the three replicates. Differences between groups were compared using analysis of variance (ANOVA) and Tukey's HSD test, with *p* ≤ 0.05 indicating significance between data.

## 3 Results and Discussion

### 3.1 Quality of Steamed Bread

The specific volume and texture of the steamed bread are important factors for evaluation of the quality of the steamed bread. The desirable qualities of steamed bread include its large size and soft texture. As shown in Table 2, the specific volume was increased after the addition of SSL and GOD, respectively. However, there was no significant difference compared with the control sample (*p* > 0.05); this may be attributed to the low levels of addition. When CM was added, the specific volume significantly increased by 9.62% (*p* ≤ 0.05). In addition, compared with the control sample, the hardness and chewiness of the SSL sample were significantly reduced by 18.66% and 16.03%, respectively (*p* < 0.05), and the corresponding values for the GOD sample were 21.67% and 16.48%, respectively (*p* ≤ 0.05). While adding CM, the hardness and chewiness of the steamed bread were reduced by 35.70% and 32.15% compared with the control (*p* ≤ 0.05). It was confirmed that the improvement effect of CM is bigger than that of a single modifier. Meanwhile, no significant change was observed in the springiness and resilience of the steamed bread, which indicated that the modifiers had no negative effect on the quality of the steamed bread. A previous relevant study also reported that the addition of 30 mg/kg of GOD caused steamed bread to have a higher specific volume and softer texture. This is because GOD enhances the strength of the gluten network and improves the dough's air‐holding capacity and uniformity (Rasiah et al. 2005). Additionally, SSL was able to form a lamellar‐structured liquid film on the interface between gluten bundles and starches, which helps to improve the air‐holding capacity of the dough (Li et al. 2013). The coaddition of GOD and SSL may have resulted in a synergistic effect, which enhances the improvement of the specific volume and texture of steamed bread by CM.

**TABLE 2:** Effects of different modifiers on the specific volume and texture properties of the steamed bread.

Samples	Specific volume (mL/g)	Hardness (g)	Chewiness	Springiness	Resilience
Control	2.39 ± 0.02^b^	2160.76 ± 28.21^a^	1614.77 ± 48.30^a^	0.93 ± 0.01^a^	0.47 ± 0.01^b^
SSL	2.49 ± 0.03^b^	1757.56 ± 13.74^b^	1356.00 ± 34.74^b^	0.94 ± 0.01^a^	0.46 ± 0.01^b^
GOD	2.56 ± 0.01^b^	1692.50 ± 46.12^c^	1348.60 ± 36.18^b^	0.95 ± 0.01^a^	0.50 ± 0.01^a^
CM	2.62 ± 0.02^a^	1389.31 ± 38.15^d^	1095.70 ± 40.04^c^	0.94 ± 0.01^a^	0.47 ± 0.00^b^

*Note:* SSL represents sodium stearoyl lactate, GOD represents glucose oxidase, and CM represents compound modifier. The significant difference (*p* ≤ 0.05) is expressed by different letters in the same column. Tukey's HSD test was employed to compare the data, with the results presented as means ± standard deviations (*n* = 3).

### 3.2 Rheological Properties

**FIGURE 1:**
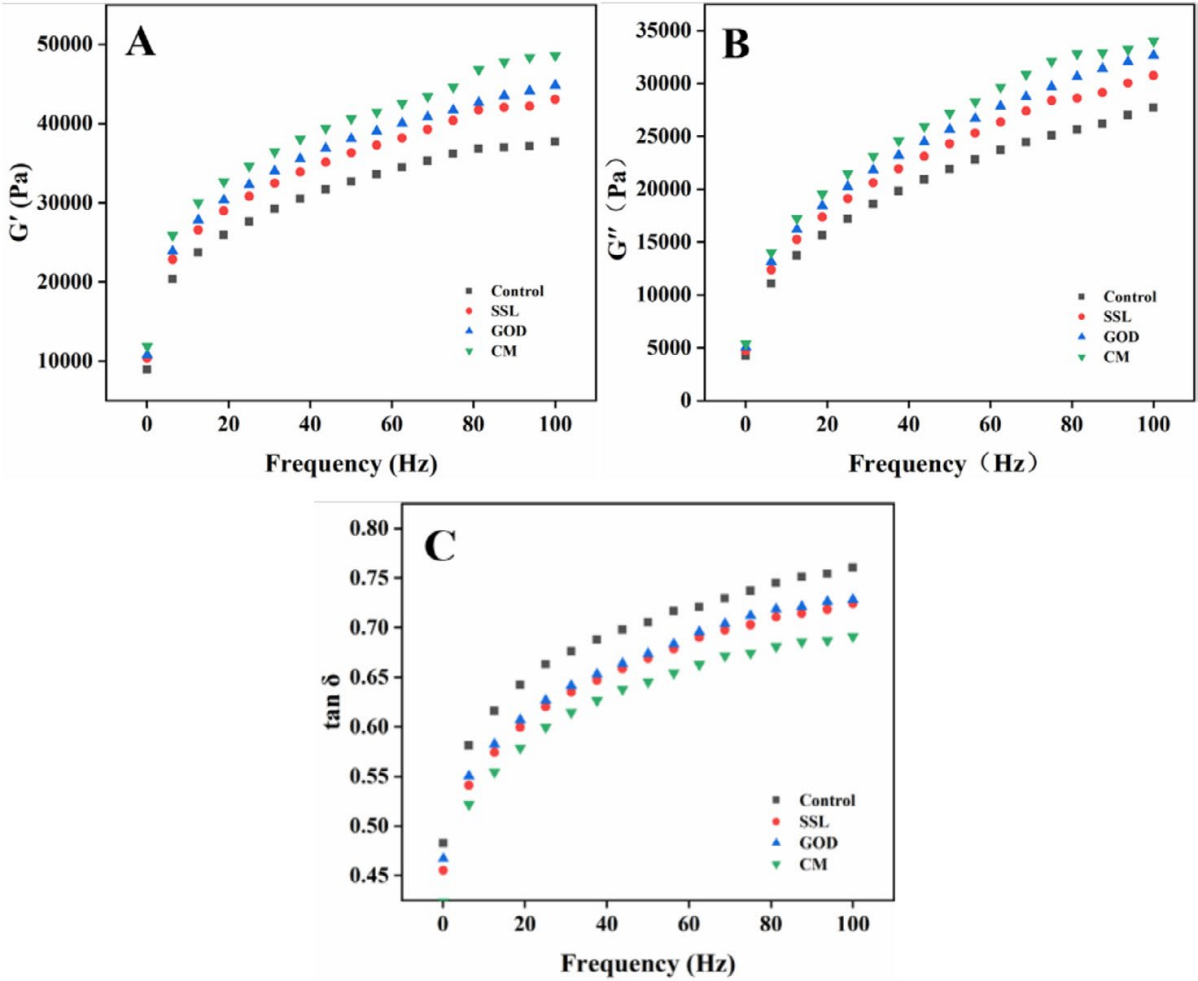
Effects of different modifiers on rheological properties of the dough. (A) elastic modulus (*G*′), (B) viscous modulus (*G*″), and (C) loss angle tangent (tan δ). SSL represents sodium stearoyl lactate, GOD represents glucose oxidase, and CM represents compound modifier.

The viscoelasticity is an important indicator to evaluate the processing properties of the dough. The storage modulus (*G*′) indicates the elastic changes of the dough, the loss modulus (*G*″) represents the viscous changes of the dough, and the loss angle tangent (tan *δ* = *G*″/*G*′) signifies the ratio of viscoelasticity of the dough. As shown in Figure 1, at the same frequency, *G*″ was always lower than *G*′ and tan *δ* was < 1, indicating that the dough was more likely to exhibit elastic properties (Li, Cao, et al. 2021; Li, Guo, et al. 2021). At the same frequency, the *G*′ and *G*″ values of doughs with different improvers added were all higher than those of the control group, indicating that the addition of improvers enhanced the rheological properties of the dough. Among them, the CM group had the highest *G*′ and *G*″ values at the same frequency, suggesting that CM had the best effect on improving the rheological properties of the dough. Interestingly, the trend of tan *δ* values was the exact opposite, which was similar to previous research findings (Zhang et al. 2024). A lower tan δ value indicates that the dough is less prone to plastic deformation under stress, enabling it to maintain structural stability during processing. At the same frequency, the control group had the highest tan *δ* value and the CM group had the lowest, indicating superior processing performance of the CM group dough. GOD, as an oxidizing enzyme, is able to catalyze the conversion of free ‐SH to ‐S‐S in the dough (Niu et al. 2018), and the ‐S‐S plays a critical role in maintaining the rheological properties of dough (Wang et al. 2018). Meanwhile, SSL can modulate the interactions between components in the dough, promoting the formation of chain‐like structures from freely dispersed glutenin, which is more conducive to forming a dense gluten network (Lu and Seetharaman 2013). The significant improvement in the rheological properties of the dough by CM may also be attributed to the synergistic effect produced by the coaddition of SSL and GOD.

### 3.3 Water Distribution

**FIGURE 2:**
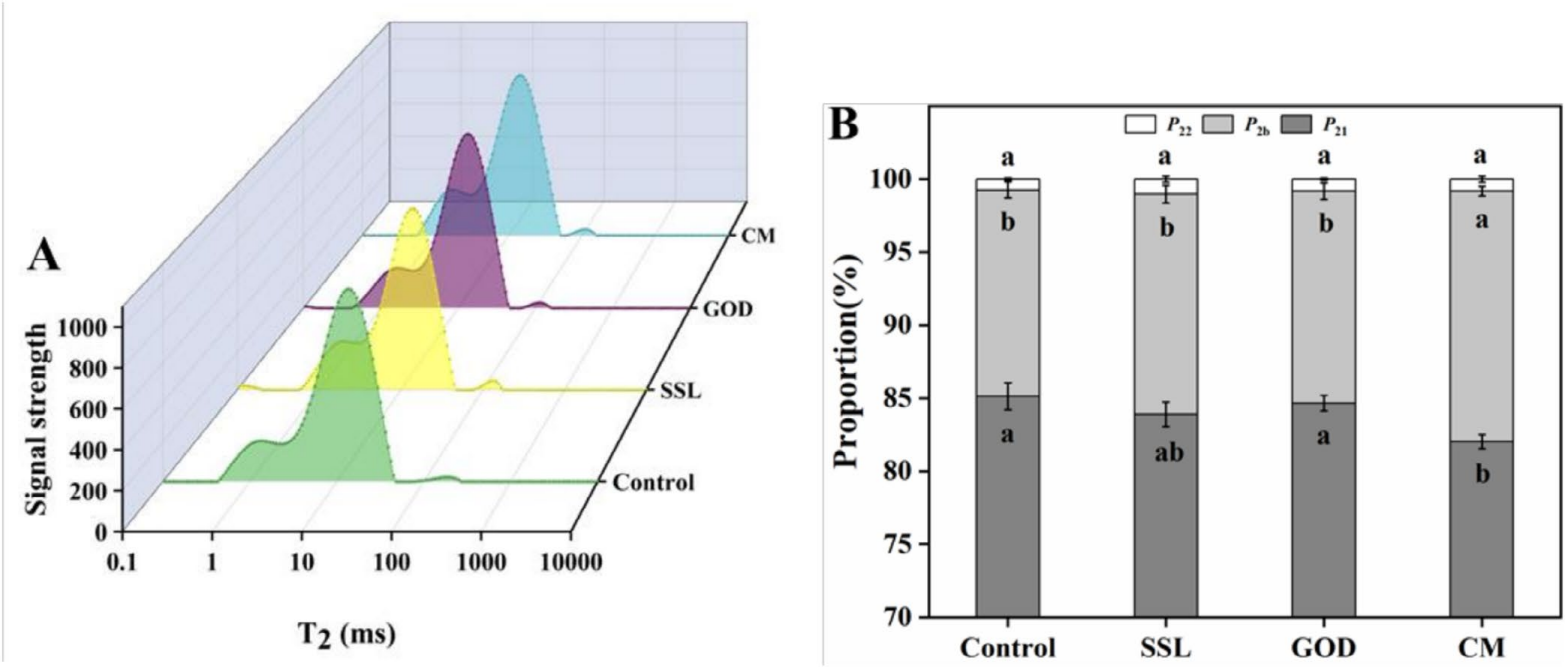
Distribution of the LF‐NMR *T*_2_ relaxation times (A) and *T*_2_ peak ratio (B) of the dough. The means with different letters in the same index differ significantly (*p* ≤ 0.05). SSL represents sodium stearoyl lactate, GOD represents glucose oxidase, and CM represents compound modifier. Tukey's HSD test was employed to compare the data, with the results presented as means and standard deviations (*n* = 3).

The content and distribution of water greatly affect the rheological and processing properties of the dough (Huang et al. 2016). Water in the dough is generally bound to the gluten–starch network, and can be classified into three types according to the closeness of binding. The three types correspond to the three peaks of the transverse relaxation time (*T*_2_) spectra (Figure 2A). *T*_2b_ (0.01–3.05 ms) reflects the water closely bound to the gluten–starch network, *T*_21_ (3.05–75 ms) represents immobilized water, and *T*_22_ (75–500 ms) corresponds to free water that can move freely in the dough (Zhu et al. 2022). As shown in Figure 2B, P_2b_, P_21_, and P_22_ represent the corresponding peak area percentage. The addition of modifiers had no significant effect on the proportion of P_22_ (*p* > 0.05), which was consistent with previous results (Lu and Seetharaman 2013). Generally speaking, the content of free water in dough is quite low; thus its changes can be neglected. In addition, there was no significant difference in the proportions of P_2b_ and P_21_ in the control, SSL, and GOD doughs (*p* > 0.05), indicating that SSL or GOD had limited influence on the water distribution in the dough. However, compared with the control sample, the addition of CM led to a significant decrease in the proportion of P_21_ (from 85.06% in the control group to 81.32% in the CM group) and a significant increase in that of P_2b_ (from 14.12% in the control group to 17.15% in the CM group) (*p* ≤ 0.05). This may be due to CM regulating the interactions among components within the dough, thereby promoting the conversion of immobilized water to bound water. SSL could modulate the relationships among water, starch, and protein in the dough (Niu et al. 2017); meanwhile, GOD can enhance the water‐binding capacity of gluten by promoting the development of disulfide bonds and protein polymerization, as the formation of a stronger gluten network requires more water (Xiao et al. 2021). The above results indicate that CM could restrict the mobility of water in the dough, thereby improving its water‐holding capacity and rheological properties.

### 3.4 Free ‐SH and ‐S‐S Contents

**FIGURE 3:**
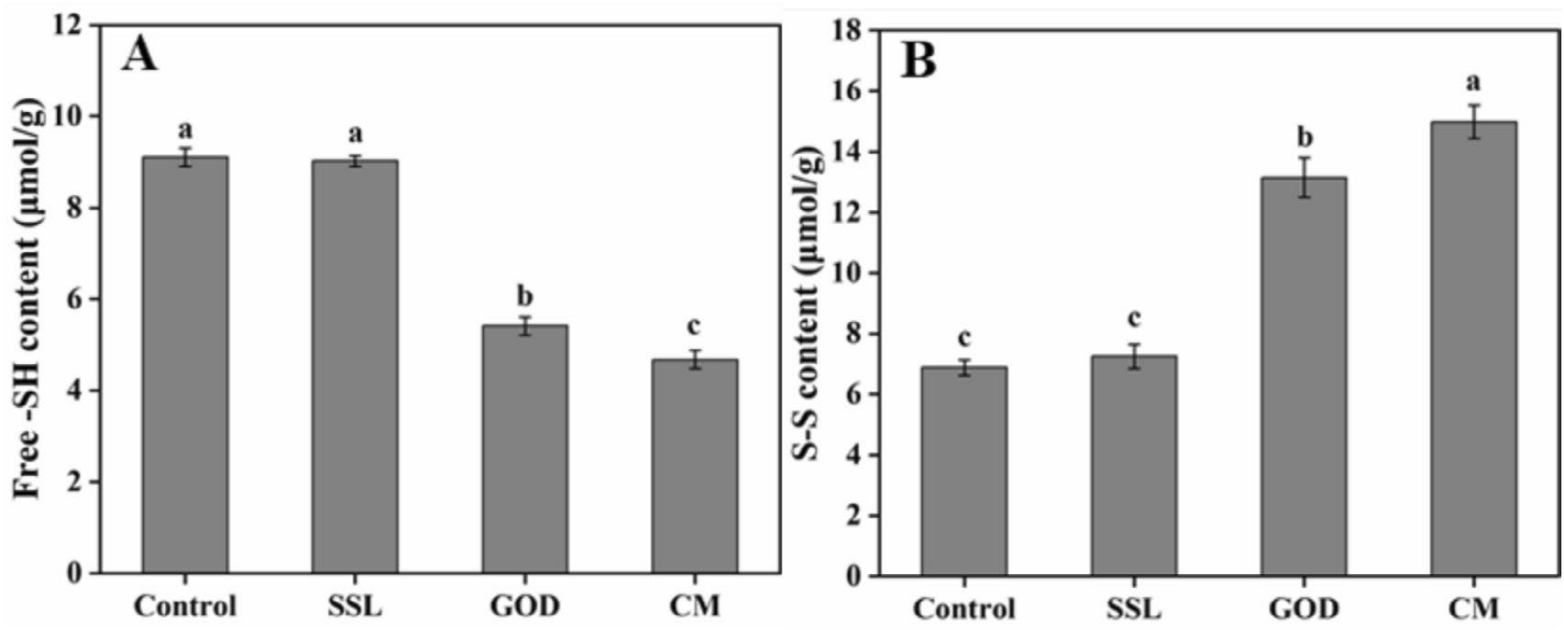
Effects of different modifiers on the content of free ‐SH (A) and ‐S‐S (B) in the dough. SSL represents sodium stearoyl lactate, GOD represents glucose oxidase, and CM represents compound modifier. The means with different letters in the same index differ significantly (*p* ≤ 0.05). Tukey's HSD test was employed to compare the data, with the results presented as means and standard deviations (*n* = 3).

Gluten is a dense, networked, elastic structure constituted by wheat proteins (Wang et al. 2021). To evaluate the effects of different modifiers on the dough gluten, the changes of free ‐SH and ‐S‐S contents in the dough were further investigated. As depicted in Figure 3, no significant difference in the free ‐SH and ‐S‐S contents was found between the SSL and control samples (*p* > 0.05). However, with the addition of GOD, the content of free ‐SH decreased by 40.54% and the ‐S‐S content increased by 90.88% compared with the control sample (*p* ≤ 0.05). The possible reason was that GOD could catalyze the conversion of glucose to gluconic acid and hydrogen peroxide in the dough, and the hydrogen peroxide further oxidizes ‐SH to form ‐S‐S. Noteworthy, the effect of CM was the most significant, resulting in a reduction of free ‐SH by 48.65% and an increase of ‐S‐S by 117.60% compared with the control sample (*p* ≤ 0.05), which may be attributed to SSL modulating the distribution of dough components, thereby exposing more free sulfhydryl groups to the action of GOD. The result confirmed that the combined effect of GOD and SSL improved the quality of the dough and steamed bread. The ‐S‐S content was usually considered to be related to the crosslinking between gluten proteins (Qian et al. 2021). The formation of disulfide bonds between gluten proteins leads to an enhancement in the pasting viscosity of wheat dough; conversely, the disruption of these disulfide bonds within the protein matrix results in a reduction of the pasting viscosity of wheat dough (Lagrain et al. 2008). The above results suggested that the addition of CM decreased ‐SH content and promoted the development of disulfide bonds, resulting in the formation of an elastic and strengthened gluten network in the dough.

### 3.5 Gluten Protein Secondary Structure

**FIGURE 4:**
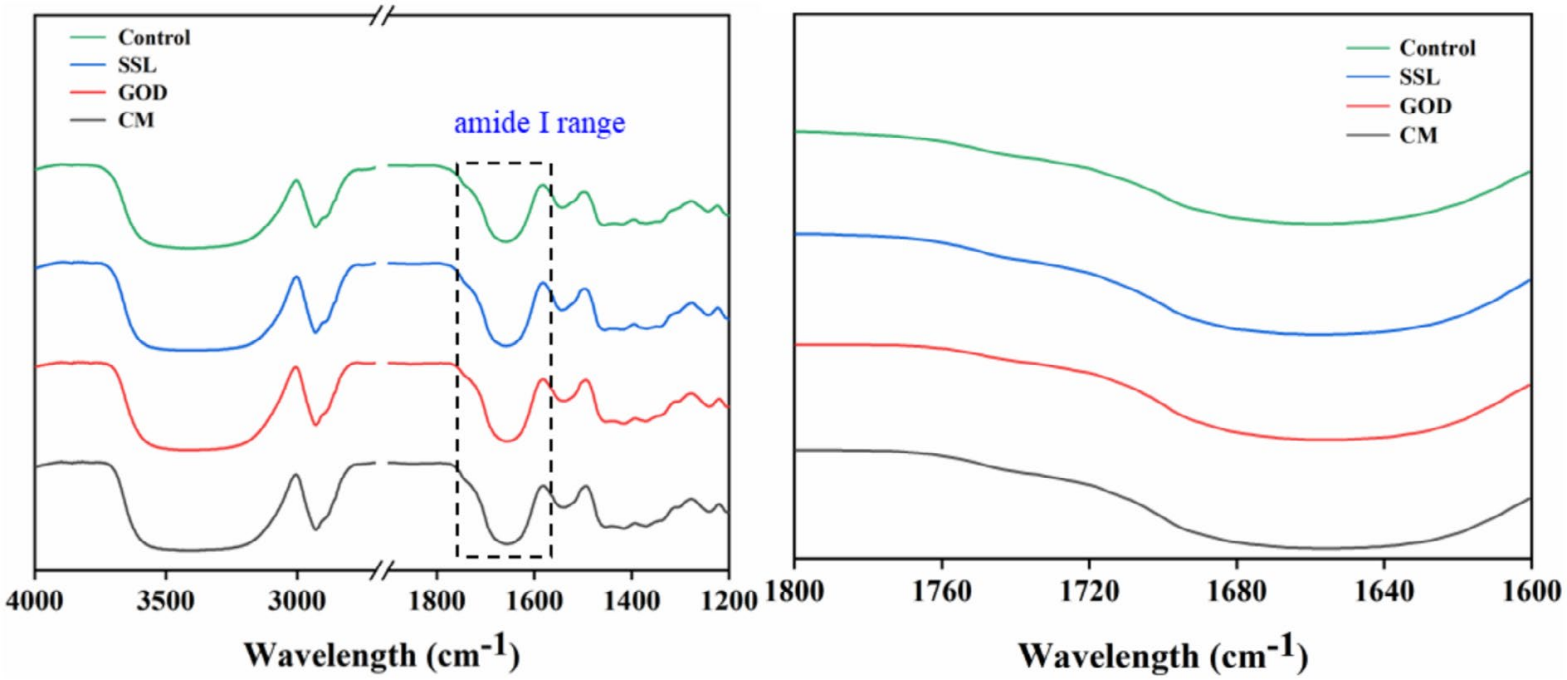
Fourier transform infrared spectra of the dough. SSL represents sodium stearoyl lactate, GOD represents glucose oxidase, and CM represents compound modifier.

To further verify the effects of different modifiers on the gluten protein structure, the FTIR spectra of dough were measured (Figure 4). The Amide I band (1600–1700 cm^−1^) of FTIR is commonly used to study the secondary structure of gluten protein. The *β*‐sheet characteristic peak is observed in the range of 1615–1637 cm^−1^ and 1682–1700 cm^−1^, while the peak in the range of 1637–1645 cm^−1^ represents the random coil structure. The *α*‐helix characteristic peak is observed in the range of 1646 to 1664 cm^−1^, and the *β*‐turn characteristic peak is present within 1664–1681 cm^−1^ (Bock and Damodaran 2013).

The peak area percentage of the secondary structure in each dough sample is summarized in Table 3. The proportion of *β*‐sheet was generally highest in all the dough samples, which indicated that *β*‐sheet is the dominant structure of gluten protein. This is consistent with the previously reported results (Nawrocka et al. 2017; Yu et al. 2020). It was clear that the proportion of β‐sheet increased significantly (*p* ≤ 0.05) with the addition of modifiers. The highest was in the CM sample, with an increase of 18.62% compared with the control sample. Additionally, the addition of modifiers resulted in a significant (*p* ≤ 0.05) decrease in β‐turn, and the lowest was also observed in the CM sample, with a 28.77% reduction compared with the control sample.

**TABLE 3:** Content of secondary structure in gluten protein.

Samples	*β*‐sheet (%)	Random coil (%)	*α*‐helix (%)	*β*‐turn (%)
Control	29.05 ± 0.01^d^	14.32 ± 0.00^b^	23.41 ± 0.00^d^	33.23 ± 0.00^a^
SSL	32.46 ± 0.02^c^	15.38 ± 0.01^a^	27.09 ± 0.01^a^	25.07 ± 0.01^b^
GOD	33.26 ± 0.01^b^	15.75 ± 0.01^a^	26.74 ± 0.01^b^	24.25 ± 0.01^c^
CM	34.46 ± 0.02^a^	15.53 ± 0.01^a^	26.33 ± 0.01^c^	23.67 ± 0.02^d^

*Note:* SSL represents sodium stearoyl lactate, GOD represents glucose oxidase, and CM represents compound modifier. The significant difference (*p* ≤ 0.05) is expressed by different letters in the same column. Tukey's HSD test was employed to compare the data, with the results presented as means ± standard deviations (*n* = 3).

Both β‐sheet and *β*‐turn are the main secondary structures of glutenin, which are directly correlated with the elastic properties of dough. Wellner et al. (2005) have reported that the dough elastic energy could be stored when *β*‐turn was converted to *β*‐sheet. Furthermore, the enhancement of the gluten network was also thought to be associated with the conversion of *β*‐turn to *β*‐sheet (Belton 1999; Hollosi et al. 1994). *β*‐sheet is considered to be positively correlated with the viscoelasticity of dough and is also known as the most stable protein conformation (Yu et al. 2020). Therefore, the decreased *β*‐turn and the increased *β*‐sheet conformation by CM indicated the development of a more elastic gluten network.

The proportion of α‐helix in the CM sample was also increased compared with the control sample (*p* ≤ 0.05), which implied a more ordered gluten network formed (Ferrer et al. 2008). However, the change in random coil structure was minimal. In sum, the addition of CM resulted in the increase of *β*‐sheet and *α*‐helix conformations, and the decrease of *β*‐turn conformation. These changes strengthened the stability of the gluten protein secondary structure, which resulted in the formation of an elastic and organized gluten network in the CM dough.

### 3.6 Microstructure of Dough and Steamed Bread

**FIGURE 5:**
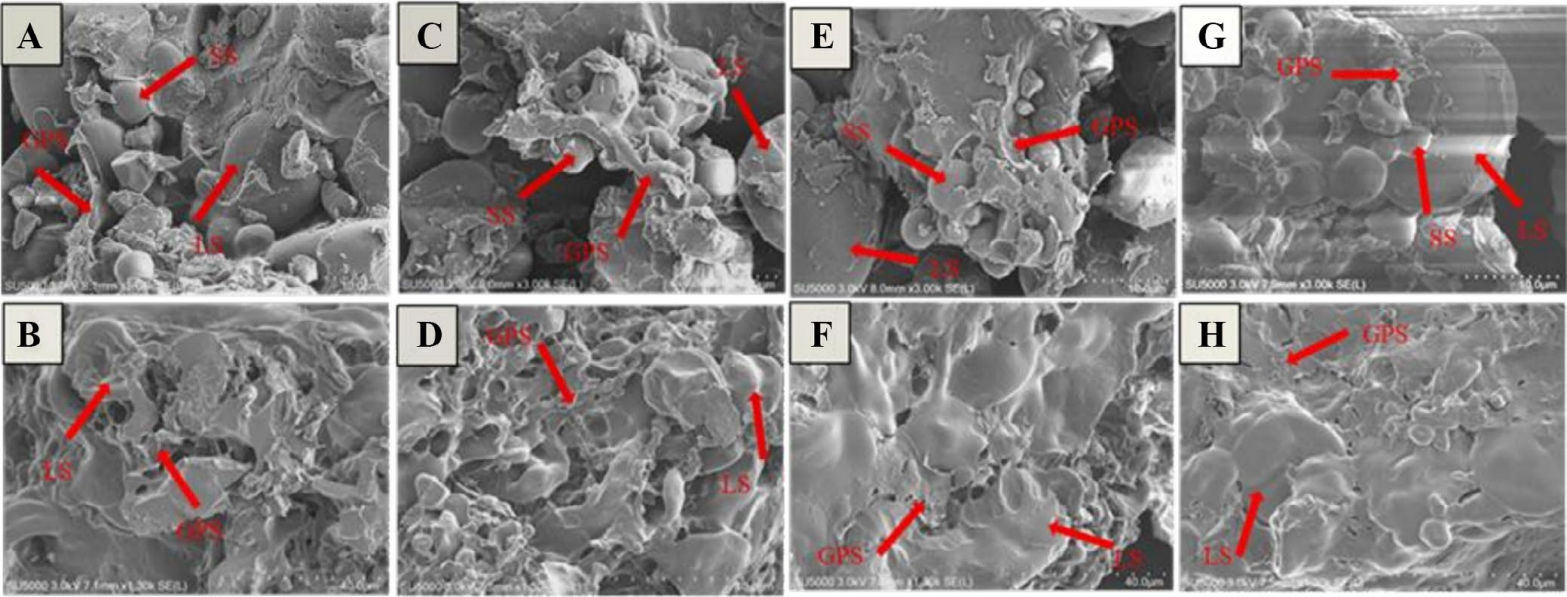
Effects of different modifiers on the microstructure of samples: (A) the dough of the control group, (B) the steamed bread of the control group, (C) the dough of the SSL group, (D) the steamed bread of the SSL group, (E) the dough of the GOD group, (F) the steamed bread of the GOD group, (G) the dough of the CM group, (H) the steamed bread of the CM group. GPS represents the gluten protein structure, LS represents the large starch, and SS represents the small starch.

Dough is a complex system in which large and small starch granules were embedded in the gluten protein network (Meng et al. 2021). Figure 5 shows the microstructures of the dough and steamed bread with different modifiers. It can be found that the starch granules showed a smooth surface without depression, which was encapsulated in the gluten network. In the control dough (Figure 5A), the starch granules were mostly exposed and not completely incorporated into the gluten matrix. Compared with the control dough, the starch granules were better incorporated into the gluten matrix in both SSL (Figure 5C) and GOD (Figure 5E) doughs, but their gluten networks were not uniform. Niu et al. (2017) also reported that the starch granule encapsulation rate increased and the compactness of the gluten network was improved in dough with added SSL or GOD, which is consistent with the present study. Surprisingly, the CM dough (Figure 5G) showed a uniform and continuous gluten network, where the starch granules were incorporated into the gluten matrix to form a continuous film. This result confirms from a microscopic perspective that CM can regulate the interactions among components in the dough, leading to the formation of a dense and continuous gluten network structure, thereby enhancing the quality of the dough (Lucas et al. 2019).

The microstructure of the steamed bread was apparently different from that of the dough. After the dough was steamed into buns, the starch granules became dehydrated and deformed. This was because the high temperature during the steaming process melted the starch granules and made them lose their original shapes (Tan et al. 2023). In the control and SSL‐steamed bread (Figure 5B,D), the starch granules were exposed on the gluten matrix, which showed flattened and depressed states. In addition, the gluten network was full of holes and lost continuity in the control steamed bread. Distinct from the steamed bread of the control and SSL, the steamed bread with the addition of GOD (Figure 5F) or CM (Figure 5H) showed an obviously smoother gluten network, with the starch granules maintaining relatively intact shapes and being wrapped by the gluten matrix. Most noteworthy, the gluten network of the CM steamed bread exhibited a more continuous surface than that of the GOD steamed bread. This may be because GOD promotes the development of the gluten network structure while SSL regulates the distribution of dough components (Niu et al. 2017, 2018). Together, they have a synergistic effect, forming a compact gluten network structure and facilitating the incorporation of starch granules into the gluten matrix.

## 4 Conclusions

In general, CM significantly improved the rheological properties of the dough as well as the texture and specific volume of the steamed bread, where CM was always more effective than the single modifier that composed it. LF‐NMR results showed that CM promoted the conversion of immobilized water to bound water, which restricted the water mobility of the dough. In addition, FTIR and sulfhydryl group determination results suggested that CM increased the content of *β*‐sheet, *α*‐helix, and ‐S‐S in the dough, which are the stable structures of the gluten protein. SEM results also demonstrated that the dough and steamed bread with CM formed a more polymerized and strengthened gluten network, with the starch granules incorporated into it.

Notably, from the results of LF‐NMR and sulfhydryl group determination, the combined improvement effect was verified to occur between GOD and SSL. We speculate that it might be the action of SSL in modulating the components of the dough that creates a better environment for GOD to exert its enzymatic activity, thereby enhancing the dough‐improving effects of GOD. In the future, the mechanism by which SSL and GOD synergistically improve dough quality could be explored by studying the direct interactions between SSL and GOD, or by measuring the effects of SSL on the enzymatic activity of GOD. All in all, these results demonstrated that GOD and SSL may serve as effective compound modifiers used in the flour products.

## Conflicts of Interest

The authors declare no conflicts of interest.

## Data Availability

The data that support the findings of this study are available from the corresponding author upon reasonable request.
